# Crystal structures of (1*E*,4*E*)-1,5-bis­(5-bromo­thio­phen-2-yl)-2,4-di­methyl­penta-1,4-dien-3-one and (*E*)-4-(5-bromo­thio­phen-2-yl)-1,3-di­phenyl­but-3-en-2-one

**DOI:** 10.1107/S205698901600058X

**Published:** 2016-01-20

**Authors:** C. Nithya, M. Sithambaresan, M. R. Prathapachandra Kurup

**Affiliations:** a Department of Applied Chemistry, Cochin University of Science and Technology, Kochi 682 022, India; b Department of Chemistry, Faculty of Science, Eastern University, Chenkalady, Sri Lanka

**Keywords:** crystal structure, Claisen–Schmidt, 5-bromo­thio­phene-2-carbaldehyde, pentan-3-one, di­benzyl­acetone

## Abstract

The title compounds, C_15_H_12_Br_2_OS_2_, (I), and C_20_H_15_BrOS, (II), were synthesized by employing Claisen–Schmidt condensation of pentan-3-one and di­benzyl­acetone with 5-bromo­thio­phene-2-carbaldehyde in the presence of methano­lic KOH. Even though 1:2 products were expected in both of the reactions, 1:2 and 1:1 products were obtained as (I) and (II), respectively. In (I), the two methyl groups are *trans* to each other, 29.5 (7) and 28.7 (7)° away from the central carbonyl bond between them, whereas the two phenyl rings of di­benzyl­acetone subtend a dihedral angle of 53.09 (18)°. In the crystal of (I), C—H⋯O hydrogen bonds define mol­ecular chains along *c*. A second type of mol­ecular chain is formed along *b* by means of C—Br⋯π inter­actions. These two families of mol­ecular chains are stacked by π–π inter­actions, forming a three-dimensional supra­molecular architecture. In (II), similar C—H⋯O hydrogen bonds as in (I) define inversion dimers, whilst C—H⋯.π inter­actions build a staircase structure along the *a* axis.

## Abstract 2

Both title structures present non-classical inter­molecular C—H⋯O, C—Br⋯π, C—H⋯π and π–π inter­actions which form three-dimensional supra­molecular architectures by means of different linkages in their crystal structures.

## Chemical context   

Claisen–Schmidt reaction (Claisen & Claparede, 1881 ▸; Schmidt, 1881 ▸) is the condensation of aromatic aldehydes (or between ketones and aldehydes lacking α-hydrogen with aliphatic or mixed alkyl aryl ketones in the presence of a relatively strong base to form α,β-unsaturated ketones. This reaction is of tremendous value in synthetic organic chemistry (Wayne & Adkins 1940 ▸; Marvel & King, 1944 ▸) and is frequently encountered as a key step in several elegant total synthesis protocols. Claisen–Schmidt condensation can also be catalysed by acid. The first step is a condensation of an aldol type; enols or enolates are involved as inter­mediates in this reaction. This reaction involves the nucleophilic addition of enol or an enolate ion derived from methyl ketone to the carbonyl carbon of the aromatic aldehyde. Dehydration of the hy­droxy­lketone to form the conjugated unsaturated carbonyl compound occurs spontaneously (see Scheme 1) (Stiles *et al.*, 1959 ▸). Cyclo­alkanones like cyclo­hexa­none, cyclo­hepta­none readily undergo Claisen–Schmidt condensation (Nithya *et al.*, 2014 ▸). In addition to cyclo­alkanones we attempted open-chain alkanones.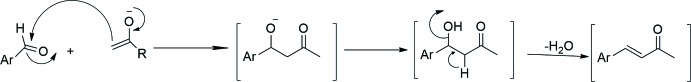 
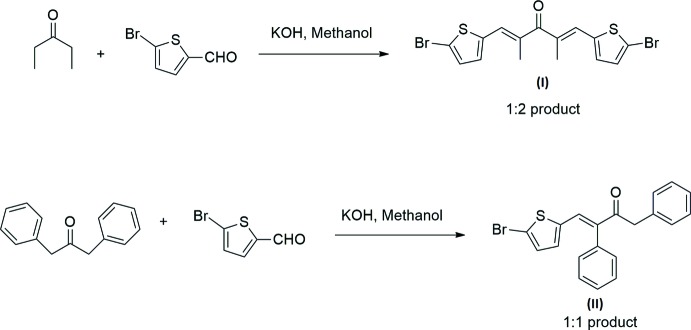


The title compounds (I) and (II) were synthesized by employing Claisen–Schmidt condensation of pentan-3-one and di­benzyl­acetone with 5-bromo­thio­phene-2-carbaldehyde in the presence of methano­lic KOH (Schemes 1 and 2). Although we anti­cipated getting 1:2 products in both of the reactions, a 1:2 product was obtained for the former case, (I), and a 1:1 product for the latter, (II). In compound (II), the bulky phenyl ring hinders the possibility of a second bromo­thio­phene ring being attached and hence only a 1:1 product was formed in this case. We present herein the structures of (1*E*,4*E*)-1,5-bis­(5-bromo­thio­phen-2-yl)-2,4-di­methyl­penta-1,4-dien-3-one (I) and (*E*)-4-(5-bromo­thio­phen-2-yl)-1,3-di­phenyl­but-3-en-2-one (II).

## Structural commentary   

**Figure 1:**
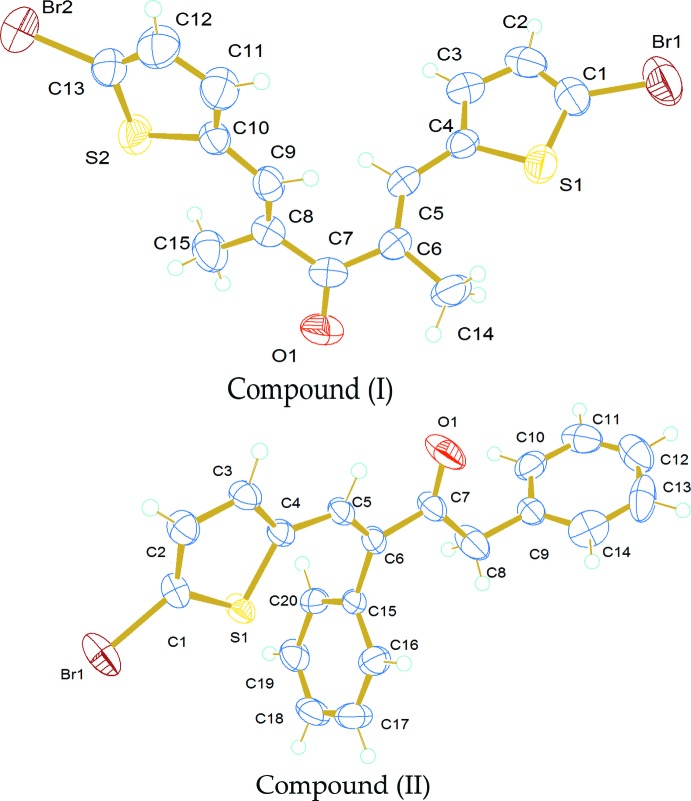
View of the title compounds (I) and (II) drawn with 50% probability displacement ellipsoids for the non-H atoms.

The mol­ecular structures of (I) and (II) are shown in Fig. 1 ▸. The asymmetric unit of (I) comprises one mol­ecule of bis(bromothiophenyl)dimethylpentanone and two of 5-bromothiophenyldiphenylbutanone. The two methyl groups (C14 and C15) of (I) are twisted away from each other with C14—C6—C7—O1 and C15—C8—C7—O1 torsion angles of 29.5 (7) and 28.7 (7)°, respectively.

The asymmetric unit of (II) comprises one mol­ecule of 5-bromo­thio­phene-2-carbaldehyde with one mol­ecule of di­benzyl­acetone. The two phenyl rings of the di­benzyl­acetone subtend a dihedral angle of 53.09 (18)°. One of the phenyl rings (C15–C20) of the di­benzyl­acetone and the thio­phene ring are normal to one another, forming a dihedral angle of 89.96 (16)°.

## Supra­molecular features   

**Figure 2:**
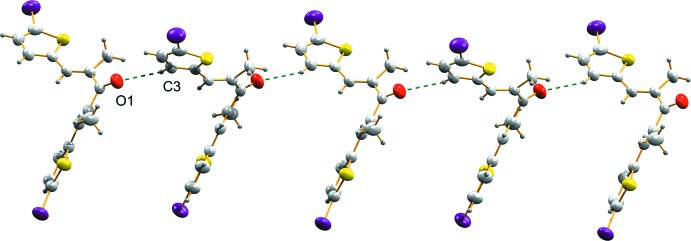
C—H⋯O hydrogen-bonding inter­action in (I), forming a mol­ecular chain along the *c* axis.

**Figure 3:**
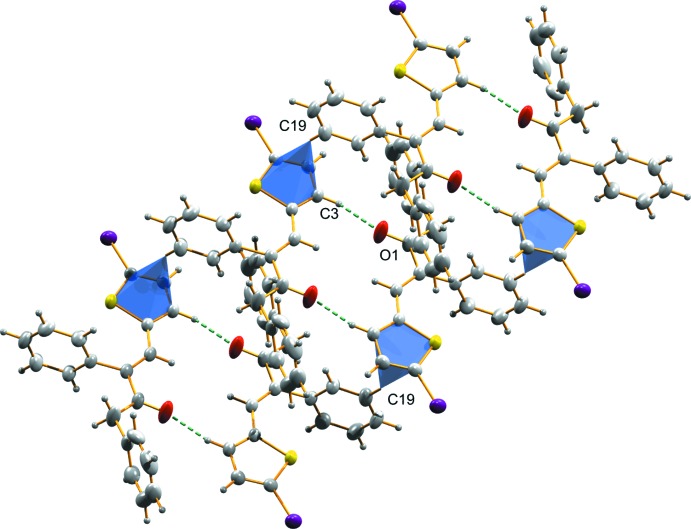
The mol­ecular chain in (I), formed along the *b* axis through C—Br⋯π inter­actions.

**Figure 4:**
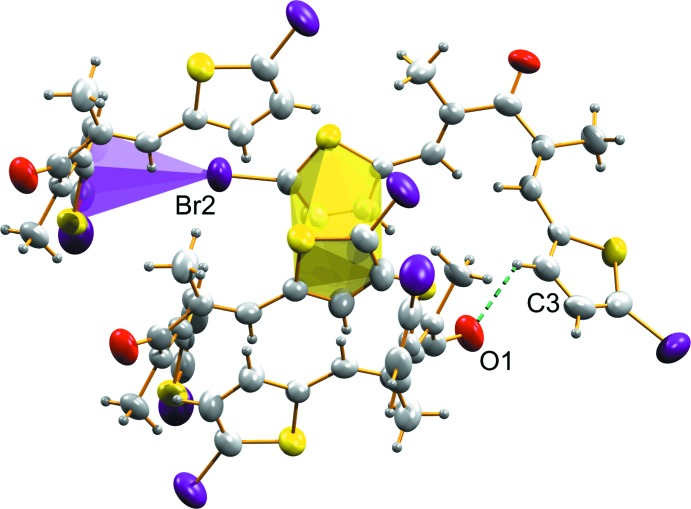
The two mol­ecular chains in (I), stacked by π–π inter­actions to form a three-dimensional supra­molecular architecture.

In the crystal structure of (I), a non-classical C—H⋯O hydrogen bond (Table 1 ▸) links the mol­ecules into a chain along the *c* axis (Fig. 2 ▸). Another mol­ecular chain is formed along the *b* axis through a C13—Br2⋯π(C1–C4/S1)^ii^ inter­action [symmetry code: (ii) 1 − *x*, 1 + *y*, 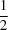 − z], Br⋯*Cg =* 3.556 (2) Å (Fig. 3 ▸). The two mol­ecular chains are in turn stacked by π–π inter­actions between the two thio­phene rings, (C1–C4/S1) and (C10–C13/S2)^iii^ [symmetry code: (iii) 1 − *x*, *y*, 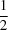 − *z*], *Cg*⋯*Cg =* 3.718 (3) Å, forming a three-dimensional supra­molecular architecture (Fig. 4 ▸).

**Figure 5:**
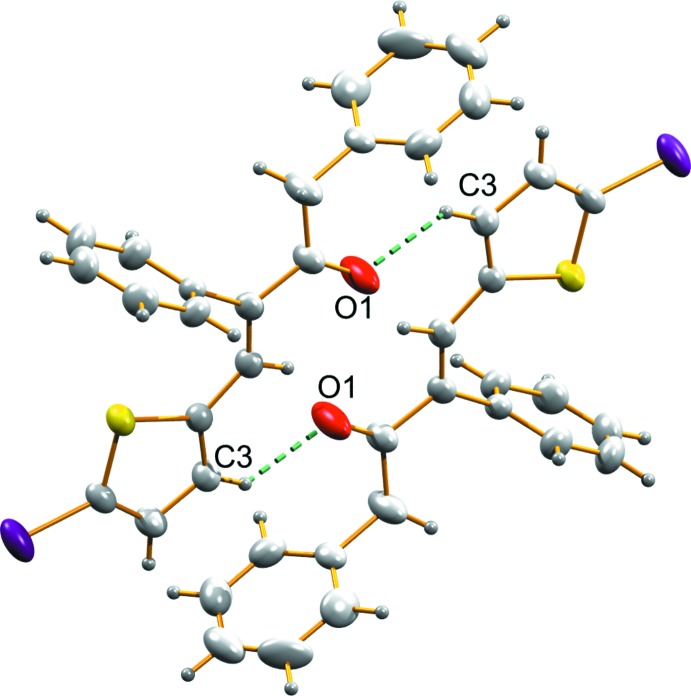
C—H⋯O inter­actions in (II), forming an inversion dimer.

**Figure 6:**
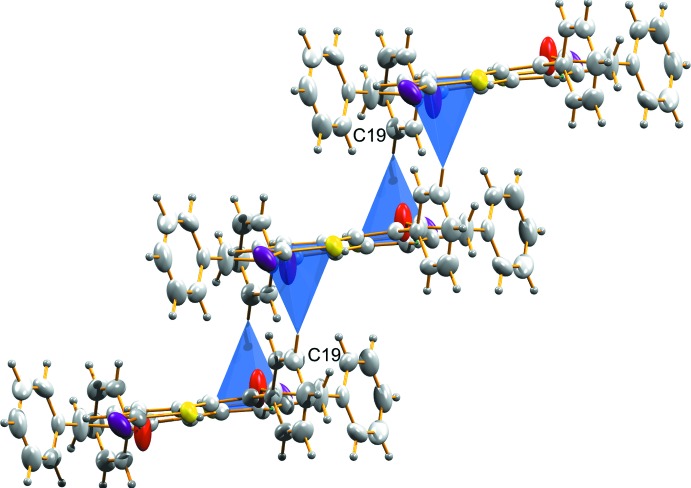
Dimers in (II) linked together by means of C—H⋯π inter­actions building a staircase structure along the *a* axis.

In structure (II) a C—H⋯O hydrogen bond (Table 2 ▸) links pairs of mol­ecules, forming inversion dimers (Fig. 5 ▸). The dimers are linked together by means of C19–H19⋯π(C1–C4/S1) inter­action, building a staircase structure along the *a* axis (Fig. 6 ▸).

## Synthesis and crystallization   

The title compounds were prepared by adapting a reported procedure (Alkskas *et al.*, 2013 ▸). Title compound (I) was prepared by adding a mixture of pentan-3-one (0.50 g, 1.2 mmol) and 5-bromothio­phene-2-carbaldehyde (2.2 g, 2.4 mmol) in methanol (25 mL) and potassium hydroxide pellets (0.2 g, 2.4 mmol) was also added. The reaction mixture was stirred at room temperature overnight whilst a pale-yellow product separated out. The crude product was washed several times with cold ethanol (1 mL). Good quality single crystals suitable for X-ray analysis were obtained by recrystallization from chloro­form, m.p. 401–403 K. Yield: 85%. IR (KBr): 1680 (C=O), 3061(=C—H). ^1^H NMR: (CDCl_3_): δ2.20 (3H, *s*), δ6.97–6.96 (1H, *d*), δ7.08–7.07 (1H, *d*), δ7.18–7.17 (1H, *s*). MS: *m*/*z* 431 (*M* ^+^); analysis calculated for C_15_H_12_Br_2_S_2_O: C: 41.69, H: 2.80, Br: 36.98, S: 14.84; found: C: 41.59, H: 2.78, Br: 36.90, S: 14.74.

Title compound (II) was prepared by mixing di­benzyl­ketone (1 g, 4.7 mmol) and 5-bromothio­phene-2-carbaldehyde (1.8 g, 9.5 mmol) in methanol (25 mL) and potassium hydroxide pellets (0.6 g, 9.5 mmol) were also added. The reaction mixture was stirred at room temperature overnight whilst a yellow product separated out. The crude product was washed several times with cold ethanol (1 mL). Good quality single crystals suitable for X-ray analysis were obtained by recrystallization from chloro­form, m.p. 383–385 K. Yield: 90%. IR (KBr): 1627 (C=O), 3080 (=C—H). ^1^H NMR (CDCl_3_): δ3.78 (2H, *s*), δ7.80 (1H, *s*), 7.51–7.48 (1H, *m*), 7.47 (1H, *m*), 7.23–7.20 (2H, *m*), 7.15–7.14 (2H, *m*), 7.13–7.12 (2H, *m*), 7.04–7.02 (2H, *m*), 6.97–6.96 (1H, *d*), 6.90–6.89 (1H, *d*). MS: *m*/*z* 383 (*M* ^+^); analysis calculated for C_20_H_15_BrOS: C: 62.67, H: 3.94, Br: 20.85, S: 8.37; found: C: 62.57, H: 3.92, Br: 20.77, S: 8.27.

## Refinement   

Crystal data, data collection and structure refinement details are summarized in Table 3 ▸. In both compounds, all H atoms on C were placed in calculated positions, guided by difference Fourier maps, with C—H bond distances of 0.93–0.97 Å. H atoms were assigned as *U*_iso_(H) = 1.2U_eq_(carrier) or 1.5U_eq_ (methyl C). Four reflections were omitted owing to bad agreement for compound (I).

## Supplementary Material

Crystal structure: contains datablock(s) I, II. DOI: 10.1107/S205698901600058X/bg2578sup1.cif

Structure factors: contains datablock(s) I. DOI: 10.1107/S205698901600058X/bg2578Isup2.hkl

Structure factors: contains datablock(s) II. DOI: 10.1107/S205698901600058X/bg2578IIsup3.hkl

Supporting information file. DOI: 10.1107/S205698901600058X/bg2578Isup4.cml

Supporting information file. DOI: 10.1107/S205698901600058X/bg2578IIsup5.cml

CCDC references: 1446725, 1446724

Additional supporting information:  crystallographic information; 3D view; checkCIF report

## Figures and Tables

**Table 1:** Hydrogen-bond geometry (Å, °) for (I)

*D*—H⋯*A*	*D*—H	H⋯*A*	*D*⋯*A*	*D*—H⋯*A*
C3—H3⋯O1^i^	0.93	2.57	3.233 (7)	129

Symmetry code: (i) 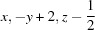.

**Table 2:** Hydrogen-bond geometry (Å, °) for (II) *Cg* is the centroid of the C1–C4/S1 ring.

*D*—H⋯*A*	*D*—H	H⋯*A*	*D*⋯*A*	*D*—H⋯*A*
C3—H3⋯O1^i^	0.93	2.54	3.320 (4)	141
C19—H19⋯*Cg* ^ii^	0.93	2.90	3.768 (3)	156

Symmetry codes: (i) 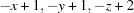; (ii) 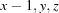.

**Table 3:** Experimental details

	(I)	(II)
Crystal data		
Chemical formula	C_15_H_12_Br_2_OS_2_	C_20_H_15_BrOS
*M*_r_	432.19	383.29
Crystal system, space group	Monoclinic, *P*2/*c*	Triclinic, *P* 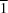
Temperature (K)	296	296
*a*, *b*, *c* (Å)	16.564 (2), 6.3581 (7), 15.962 (2)	7.5879 (4), 8.5361 (6), 14.0970 (8)
α, β, γ (°)	90, 105.239 (5), 90	99.510 (3), 97.673 (3), 101.956 (3)
*V* (Å^3^)	1622.0 (4)	867.58 (9)
*Z*	4	2
Radiation type	Mo *K*α	Mo *K*α
μ (mm^−1^)	5.25	2.49
Crystal size (mm)	0.60 × 0.50 × 0.40	0.60 × 0.50 × 0.35

Data collection		
Diffractometer	Bruker Kappa APEXII CCD	Bruker Kappa APEXII CCD
Absorption correction	Multi-scan (*SADABS*; Bruker, 2004 ▸)	Multi-scan (*SADABS*; Bruker, 2004 ▸)
*T*_min_, *T*_max_	0.049, 0.115	0.307, 0.456
No. of measured, independent and observed [*I* > 2σ(*I*)] reflections	13847, 4051, 2012	6863, 4363, 3040
*R*_int_	0.059	0.025
(sin θ/λ)_max_ (Å^−1^)	0.667	0.670

Refinement		
*R*[*F* ^2^ > 2σ(*F* ^2^)], *wR*(*F* ^2^), *S*	0.059, 0.181, 0.98	0.043, 0.118, 1.05
No. of reflections	4042	4363
No. of parameters	183	208
H-atom treatment	H-atom parameters constrained	H-atom parameters constrained
Δρ_max_, Δρ_min_ (e Å^−3^)	0.88, −0.80	0.56, −0.86

Computer programs: *APEX2*, *SAINT* and *XPREP* (Bruker, 2004 ▸), *SHELXS97* and *SHELXL97* (Sheldrick, 2008 ▸), *SHELXL2014* (Sheldrick, 2015 ▸), *ORTEP-3 for Windows* (Farrugia, 2012 ▸), *DIAMOND* (Brandenburg, 2010 ▸) and *publCIF* (Westrip, 2010 ▸).

## supplementary crystallographic information

### (I) (1E,4E)-1,5-Bis(5-bromothiophen-2-yl)-2,4-dimethylpenta-1,4-dien-3-one Crystal data

**Table:**

C_15_H_12_Br_2_OS_2_	*F*(000) = 848
*M**_r_* = 432.19	*D*_x_ = 1.770 Mg m^−^^3^
Monoclinic, *P*2/*c*	Mo *K*α radiation, λ = 0.71073 Å
*a* = 16.564 (2) Å	Cell parameters from 3021 reflections
*b* = 6.3581 (7) Å	θ = 2.6–23.4°
*c* = 15.962 (2) Å	µ = 5.25 mm^−^^1^
β = 105.239 (5)°	*T* = 296 K
*V* = 1622.0 (4) Å^3^	Block, yellow
*Z* = 4	0.60 × 0.50 × 0.40 mm

### (I) (1E,4E)-1,5-Bis(5-bromothiophen-2-yl)-2,4-dimethylpenta-1,4-dien-3-one Data collection

**Table:**

Bruker Kappa APEXII CCD diffractometer	4051 independent reflections
Radiation source: fine-focus sealed tube	2012 reflections with *I* > 2σ(*I*)
Graphite monochromator	*R*_int_ = 0.059
ω and φ scan	θ_max_ = 28.3°, θ_min_ = 2.7°
Absorption correction: multi-scan (*SADABS*; Bruker, 2004)	*h* = −22→21
*T*_min_ = 0.049, *T*_max_ = 0.115	*k* = −5→8
13847 measured reflections	*l* = −21→21

### (I) (1E,4E)-1,5-Bis(5-bromothiophen-2-yl)-2,4-dimethylpenta-1,4-dien-3-one Refinement

**Table:**

Refinement on *F*^2^	0 restraints
Least-squares matrix: full	Hydrogen site location: inferred from neighbouring sites
*R*[*F*^2^ > 2σ(*F*^2^)] = 0.059	H-atom parameters constrained
*wR*(*F*^2^) = 0.181	*w* = 1/[σ^2^(*F*_o_^2^) + (0.0913*P*)^2^ + 0.2774*P*] where *P* = (*F*_o_^2^ + 2*F*_c_^2^)/3
*S* = 0.98	(Δ/σ)_max_ = 0.001
4042 reflections	Δρ_max_ = 0.88 e Å^−^^3^
183 parameters	Δρ_min_ = −0.80 e Å^−^^3^

### (I) (1E,4E)-1,5-Bis(5-bromothiophen-2-yl)-2,4-dimethylpenta-1,4-dien-3-one Special details

**Table:**

Geometry. All e.s.d.'s (except the e.s.d. in the dihedral angle between two l.s. planes) are estimated using the full covariance matrix. The cell e.s.d.'s are taken into account individually in the estimation of e.s.d.'s in distances, angles and torsion angles; correlations between e.s.d.'s in cell parameters are only used when they are defined by crystal symmetry. An approximate (isotropic) treatment of cell e.s.d.'s is used for estimating e.s.d.'s involving l.s. planes.

### (I) (1E,4E)-1,5-Bis(5-bromothiophen-2-yl)-2,4-dimethylpenta-1,4-dien-3-one Fractional atomic coordinates and isotropic or equivalent isotropic displacement parameters (Å^2^)

**Table:**

	*x*	*y*	*z*	*U*_iso_*/*U*_eq_	
C1	0.0832 (3)	0.4182 (8)	0.1493 (4)	0.0604 (15)	
C2	0.1111 (4)	0.5540 (9)	0.0976 (4)	0.0642 (15)	
H2	0.1009	0.5392	0.0377	0.077*	
C3	0.1566 (3)	0.7172 (8)	0.1450 (4)	0.0578 (15)	
H3	0.1809	0.8227	0.1195	0.069*	
C4	0.1631 (3)	0.7115 (7)	0.2316 (3)	0.0455 (12)	
C5	0.2056 (3)	0.8689 (7)	0.2940 (3)	0.0462 (12)	
H5	0.2398	0.9604	0.2733	0.055*	
C6	0.2036 (3)	0.9036 (7)	0.3762 (3)	0.0453 (12)	
C7	0.2488 (3)	1.0879 (8)	0.4230 (3)	0.0505 (12)	
C8	0.3296 (3)	1.1597 (7)	0.4070 (3)	0.0466 (12)	
C9	0.3833 (3)	1.0165 (7)	0.3931 (3)	0.0456 (12)	
H9	0.3643	0.8784	0.3904	0.055*	
C10	0.4668 (3)	1.0418 (7)	0.3816 (3)	0.0435 (11)	
C11	0.5166 (3)	0.8833 (8)	0.3700 (4)	0.0636 (16)	
H11	0.5000	0.7433	0.3691	0.076*	
C12	0.5950 (3)	0.9419 (10)	0.3595 (4)	0.0687 (17)	
H12	0.6358	0.8485	0.3522	0.082*	
C13	0.6027 (3)	1.1518 (9)	0.3616 (4)	0.0572 (14)	
C14	0.1503 (4)	0.7812 (9)	0.4221 (4)	0.0624 (15)	
H14A	0.1493	0.8519	0.4750	0.094*	
H14B	0.1731	0.6426	0.4353	0.094*	
H14C	0.0943	0.7709	0.3854	0.094*	
C15	0.3465 (4)	1.3911 (8)	0.4187 (4)	0.0690 (17)	
H15A	0.3019	1.4570	0.4371	0.104*	
H15B	0.3500	1.4514	0.3646	0.104*	
H15C	0.3984	1.4128	0.4619	0.104*	
S1	0.11240 (8)	0.4921 (2)	0.25627 (10)	0.0591 (4)	
S2	0.51728 (9)	1.2768 (2)	0.37862 (11)	0.0610 (4)	
Br2	0.69420 (4)	1.31159 (11)	0.34996 (5)	0.0869 (3)	
Br1	0.01821 (5)	0.17864 (10)	0.11612 (6)	0.0937 (3)	
O1	0.2192 (3)	1.1834 (6)	0.4736 (3)	0.0749 (12)	

### (I) (1E,4E)-1,5-Bis(5-bromothiophen-2-yl)-2,4-dimethylpenta-1,4-dien-3-one Atomic displacement parameters (Å^2^)

**Table:**

	*U*^11^	*U*^22^	*U*^33^	*U*^12^	*U*^13^	*U*^23^
C1	0.044 (3)	0.057 (3)	0.077 (4)	0.004 (3)	0.009 (3)	−0.013 (3)
C2	0.065 (4)	0.077 (4)	0.050 (4)	0.009 (3)	0.014 (3)	−0.016 (3)
C3	0.060 (3)	0.063 (3)	0.055 (4)	0.004 (3)	0.025 (3)	0.002 (3)
C4	0.039 (3)	0.054 (3)	0.047 (3)	0.001 (2)	0.017 (2)	−0.003 (2)
C5	0.045 (3)	0.052 (3)	0.046 (3)	−0.002 (2)	0.020 (2)	0.005 (2)
C6	0.044 (3)	0.047 (3)	0.048 (3)	0.000 (2)	0.018 (2)	0.004 (2)
C7	0.057 (3)	0.057 (3)	0.039 (3)	0.007 (2)	0.015 (3)	−0.002 (3)
C8	0.049 (3)	0.047 (3)	0.042 (3)	0.001 (2)	0.012 (2)	−0.004 (2)
C9	0.052 (3)	0.039 (2)	0.044 (3)	−0.006 (2)	0.009 (2)	0.002 (2)
C10	0.048 (3)	0.038 (2)	0.045 (3)	−0.004 (2)	0.012 (2)	−0.004 (2)
C11	0.058 (3)	0.046 (3)	0.088 (5)	0.003 (2)	0.023 (3)	−0.003 (3)
C12	0.054 (3)	0.067 (4)	0.087 (5)	0.009 (3)	0.022 (3)	−0.004 (3)
C13	0.050 (3)	0.068 (4)	0.053 (4)	−0.009 (3)	0.013 (3)	−0.006 (3)
C14	0.064 (4)	0.081 (4)	0.050 (4)	−0.005 (3)	0.029 (3)	0.005 (3)
C15	0.062 (4)	0.050 (3)	0.094 (5)	0.000 (3)	0.020 (3)	−0.019 (3)
S1	0.0566 (8)	0.0638 (8)	0.0565 (10)	−0.0148 (7)	0.0141 (7)	−0.0001 (7)
S2	0.0586 (8)	0.0455 (7)	0.0828 (12)	−0.0091 (6)	0.0255 (8)	−0.0096 (7)
Br2	0.0666 (4)	0.1003 (6)	0.1022 (6)	−0.0286 (4)	0.0372 (4)	−0.0157 (4)
Br1	0.0836 (5)	0.0756 (5)	0.1146 (7)	−0.0153 (3)	0.0128 (4)	−0.0333 (4)
O1	0.077 (3)	0.087 (3)	0.069 (3)	0.000 (2)	0.036 (2)	−0.028 (2)

### (I) (1E,4E)-1,5-Bis(5-bromothiophen-2-yl)-2,4-dimethylpenta-1,4-dien-3-one Geometric parameters (Å, º)

**Table:**

C1—C2	1.357 (8)	C9—C10	1.450 (6)
C1—S1	1.714 (6)	C9—H9	0.9300
C1—Br1	1.860 (5)	C10—C11	1.346 (7)
C2—C3	1.385 (7)	C10—S2	1.720 (4)
C2—H2	0.9300	C11—C12	1.403 (8)
C3—C4	1.358 (7)	C11—H11	0.9300
C3—H3	0.9300	C12—C13	1.340 (8)
C4—C5	1.456 (7)	C12—H12	0.9300
C4—S1	1.727 (5)	C13—S2	1.705 (6)
C5—C6	1.340 (7)	C13—Br2	1.874 (5)
C5—H5	0.9300	C14—H14A	0.9600
C6—C7	1.483 (7)	C14—H14B	0.9600
C6—C14	1.505 (7)	C14—H14C	0.9600
C7—O1	1.213 (6)	C15—H15A	0.9600
C7—C8	1.497 (7)	C15—H15B	0.9600
C8—C9	1.332 (6)	C15—H15C	0.9600
C8—C15	1.500 (7)		
			
C2—C1—S1	112.3 (4)	C11—C10—C9	125.0 (4)
C2—C1—Br1	127.7 (5)	C11—C10—S2	109.1 (4)
S1—C1—Br1	119.9 (4)	C9—C10—S2	125.9 (3)
C1—C2—C3	111.6 (5)	C10—C11—C12	115.9 (5)
C1—C2—H2	124.2	C10—C11—H11	122.0
C3—C2—H2	124.2	C12—C11—H11	122.0
C4—C3—C2	115.0 (5)	C13—C12—C11	110.3 (5)
C4—C3—H3	122.5	C13—C12—H12	124.9
C2—C3—H3	122.5	C11—C12—H12	124.9
C3—C4—C5	124.9 (5)	C12—C13—S2	113.0 (4)
C3—C4—S1	109.9 (4)	C12—C13—Br2	127.7 (4)
C5—C4—S1	125.2 (4)	S2—C13—Br2	119.3 (3)
C6—C5—C4	130.6 (4)	C6—C14—H14A	109.5
C6—C5—H5	114.7	C6—C14—H14B	109.5
C4—C5—H5	114.7	H14A—C14—H14B	109.5
C5—C6—C7	119.0 (4)	C6—C14—H14C	109.5
C5—C6—C14	124.1 (5)	H14A—C14—H14C	109.5
C7—C6—C14	116.5 (4)	H14B—C14—H14C	109.5
O1—C7—C6	119.5 (5)	C8—C15—H15A	109.5
O1—C7—C8	119.8 (5)	C8—C15—H15B	109.5
C6—C7—C8	120.7 (4)	H15A—C15—H15B	109.5
C9—C8—C7	119.1 (4)	C8—C15—H15C	109.5
C9—C8—C15	125.4 (5)	H15A—C15—H15C	109.5
C7—C8—C15	115.0 (4)	H15B—C15—H15C	109.5
C8—C9—C10	130.3 (4)	C1—S1—C4	91.2 (3)
C8—C9—H9	114.9	C13—S2—C10	91.7 (2)
C10—C9—H9	114.9		
			
S1—C1—C2—C3	0.4 (6)	C7—C8—C9—C10	−175.6 (5)
Br1—C1—C2—C3	178.9 (4)	C15—C8—C9—C10	−3.3 (9)
C1—C2—C3—C4	−1.0 (7)	C8—C9—C10—C11	178.2 (6)
C2—C3—C4—C5	−178.1 (5)	C8—C9—C10—S2	−3.0 (8)
C2—C3—C4—S1	1.2 (6)	C9—C10—C11—C12	179.6 (5)
C3—C4—C5—C6	166.0 (5)	S2—C10—C11—C12	0.6 (7)
S1—C4—C5—C6	−13.1 (8)	C10—C11—C12—C13	−1.3 (8)
C4—C5—C6—C7	−175.4 (5)	C11—C12—C13—S2	1.4 (7)
C4—C5—C6—C14	−2.5 (8)	C11—C12—C13—Br2	−179.5 (4)
C5—C6—C7—O1	143.9 (5)	C2—C1—S1—C4	0.2 (4)
C14—C6—C7—O1	−29.5 (7)	Br1—C1—S1—C4	−178.4 (3)
C5—C6—C7—C8	−34.8 (7)	C3—C4—S1—C1	−0.8 (4)
C14—C6—C7—C8	151.8 (5)	C5—C4—S1—C1	178.5 (4)
O1—C7—C8—C9	144.4 (5)	C12—C13—S2—C10	−1.0 (5)
C6—C7—C8—C9	−36.9 (7)	Br2—C13—S2—C10	179.9 (3)
O1—C7—C8—C15	−28.7 (7)	C11—C10—S2—C13	0.2 (5)
C6—C7—C8—C15	150.0 (5)	C9—C10—S2—C13	−178.7 (5)

### (I) (1E,4E)-1,5-Bis(5-bromothiophen-2-yl)-2,4-dimethylpenta-1,4-dien-3-one Hydrogen-bond geometry (Å, º)

**Table:**

*D*—H···*A*	*D*—H	H···*A*	*D*···*A*	*D*—H···*A*
C3—H3···O1^i^	0.93	2.57	3.233 (7)	129

Symmetry code: (i) *x*, −*y*+2, *z*−1/2.

### (II) (E)-4-(5-Bromothiophen-2-yl)-1,3-diphenylbut-3-en-2-one Crystal data

**Table:**

C_20_H_15_BrOS	*Z* = 2
*M**_r_* = 383.29	*F*(000) = 388
Triclinic, *P*1	*D*_x_ = 1.467 Mg m^−^^3^
*a* = 7.5879 (4) Å	Mo *K*α radiation, λ = 0.71073 Å
*b* = 8.5361 (6) Å	Cell parameters from 3038 reflections
*c* = 14.0970 (8) Å	θ = 2.5–28.2°
α = 99.510 (3)°	µ = 2.49 mm^−^^1^
β = 97.673 (3)°	*T* = 296 K
γ = 101.956 (3)°	Block, yellow
*V* = 867.58 (9) Å^3^	0.60 × 0.50 × 0.35 mm

### (II) (E)-4-(5-Bromothiophen-2-yl)-1,3-diphenylbut-3-en-2-one Data collection

**Table:**

Bruker Kappa APEXII CCD diffractometer	4363 independent reflections
Radiation source: fine-focus sealed tube	3040 reflections with *I* > 2σ(*I*)
Graphite monochromator	*R*_int_ = 0.025
ω and φ scan	θ_max_ = 28.4°, θ_min_ = 2.5°
Absorption correction: multi-scan (*SADABS*; Bruker, 2004)	*h* = −10→8
*T*_min_ = 0.307, *T*_max_ = 0.456	*k* = −7→11
6863 measured reflections	*l* = −18→18

### (II) (E)-4-(5-Bromothiophen-2-yl)-1,3-diphenylbut-3-en-2-one Refinement

**Table:**

Refinement on *F*^2^	Primary atom site location: structure-invariant direct methods
Least-squares matrix: full	Secondary atom site location: difference Fourier map
*R*[*F*^2^ > 2σ(*F*^2^)] = 0.043	Hydrogen site location: inferred from neighbouring sites
*wR*(*F*^2^) = 0.118	H-atom parameters constrained
*S* = 1.05	*w* = 1/[σ^2^(*F*_o_^2^) + (0.0571*P*)^2^ + 0.3404*P*] where *P* = (*F*_o_^2^ + 2*F*_c_^2^)/3
4363 reflections	(Δ/σ)_max_ = 0.001
208 parameters	Δρ_max_ = 0.56 e Å^−^^3^
0 restraints	Δρ_min_ = −0.86 e Å^−^^3^

### (II) (E)-4-(5-Bromothiophen-2-yl)-1,3-diphenylbut-3-en-2-one Special details

**Table:**

Geometry. All e.s.d.'s (except the e.s.d. in the dihedral angle between two l.s. planes) are estimated using the full covariance matrix. The cell e.s.d.'s are taken into account individually in the estimation of e.s.d.'s in distances, angles and torsion angles; correlations between e.s.d.'s in cell parameters are only used when they are defined by crystal symmetry. An approximate (isotropic) treatment of cell e.s.d.'s is used for estimating e.s.d.'s involving l.s. planes.

### (II) (E)-4-(5-Bromothiophen-2-yl)-1,3-diphenylbut-3-en-2-one Fractional atomic coordinates and isotropic or equivalent isotropic displacement parameters (Å^2^)

**Table:**

	*x*	*y*	*z*	*U*_iso_*/*U*_eq_	
C1	0.2982 (4)	0.1132 (4)	0.6105 (2)	0.0364 (6)	
C2	0.4114 (4)	0.0812 (4)	0.6841 (2)	0.0415 (7)	
H2	0.4737	−0.0019	0.6772	0.050*	
C3	0.4219 (4)	0.1897 (4)	0.7720 (2)	0.0382 (7)	
H3	0.4943	0.1863	0.8299	0.046*	
C4	0.3170 (4)	0.3012 (3)	0.76546 (19)	0.0316 (6)	
C5	0.3029 (4)	0.4239 (4)	0.84553 (19)	0.0337 (6)	
H5	0.3787	0.4288	0.9040	0.040*	
C6	0.1973 (4)	0.5323 (3)	0.84900 (19)	0.0333 (6)	
C7	0.2176 (4)	0.6463 (4)	0.9441 (2)	0.0438 (7)	
C8	0.1294 (6)	0.7885 (5)	0.9442 (3)	0.0665 (12)	
H8A	0.1660	0.8438	0.8927	0.080*	
H8B	−0.0022	0.7469	0.9287	0.080*	
C9	0.1747 (4)	0.9109 (4)	1.0380 (2)	0.0388 (7)	
C10	0.0910 (5)	0.8834 (4)	1.1161 (3)	0.0503 (8)	
H10	0.0046	0.7861	1.1105	0.060*	
C11	0.1308 (6)	0.9941 (6)	1.2013 (3)	0.0640 (11)	
H11	0.0704	0.9724	1.2526	0.077*	
C12	0.2550 (6)	1.1327 (6)	1.2122 (3)	0.0657 (11)	
H12	0.2831	1.2062	1.2717	0.079*	
C13	0.3413 (5)	1.1690 (5)	1.1382 (4)	0.0695 (12)	
H13	0.4260	1.2680	1.1461	0.083*	
C14	0.3023 (5)	1.0560 (5)	1.0490 (3)	0.0585 (10)	
H14	0.3624	1.0792	0.9978	0.070*	
C15	0.0666 (4)	0.5442 (3)	0.76278 (18)	0.0319 (6)	
C16	0.1236 (4)	0.6394 (4)	0.6970 (2)	0.0449 (7)	
H16	0.2433	0.7016	0.7084	0.054*	
C17	0.0051 (5)	0.6430 (5)	0.6150 (2)	0.0562 (9)	
H17	0.0446	0.7085	0.5718	0.067*	
C18	−0.1706 (5)	0.5505 (5)	0.5968 (2)	0.0541 (9)	
H18	−0.2493	0.5506	0.5402	0.065*	
C19	−0.2302 (4)	0.4582 (4)	0.6615 (2)	0.0487 (8)	
H19	−0.3504	0.3971	0.6497	0.058*	
C20	−0.1119 (4)	0.4551 (4)	0.7449 (2)	0.0392 (7)	
H20	−0.1535	0.3925	0.7891	0.047*	
O1	0.3037 (4)	0.6264 (4)	1.01810 (17)	0.0792 (10)	
S1	0.20165 (10)	0.27183 (10)	0.64654 (5)	0.03775 (18)	
Br1	0.24497 (5)	0.00369 (5)	0.48059 (2)	0.05710 (15)	

### (II) (E)-4-(5-Bromothiophen-2-yl)-1,3-diphenylbut-3-en-2-one Atomic displacement parameters (Å^2^)

**Table:**

	*U*^11^	*U*^22^	*U*^33^	*U*^12^	*U*^13^	*U*^23^
C1	0.0317 (14)	0.0387 (17)	0.0340 (14)	0.0078 (12)	0.0032 (11)	−0.0036 (12)
C2	0.0421 (17)	0.0437 (18)	0.0401 (15)	0.0192 (14)	0.0055 (13)	0.0022 (13)
C3	0.0386 (15)	0.0444 (18)	0.0317 (13)	0.0158 (13)	−0.0006 (11)	0.0060 (12)
C4	0.0297 (13)	0.0339 (15)	0.0284 (12)	0.0067 (11)	−0.0008 (10)	0.0043 (11)
C5	0.0323 (14)	0.0356 (16)	0.0283 (12)	0.0059 (12)	−0.0033 (10)	0.0020 (11)
C6	0.0334 (14)	0.0305 (15)	0.0306 (13)	0.0051 (12)	−0.0027 (11)	0.0006 (11)
C7	0.0514 (18)	0.0439 (18)	0.0333 (14)	0.0233 (15)	−0.0089 (13)	−0.0029 (13)
C8	0.090 (3)	0.063 (2)	0.0447 (18)	0.050 (2)	−0.0185 (18)	−0.0109 (17)
C9	0.0452 (17)	0.0361 (17)	0.0362 (14)	0.0227 (14)	−0.0028 (12)	0.0013 (12)
C10	0.053 (2)	0.0428 (19)	0.059 (2)	0.0140 (16)	0.0056 (16)	0.0203 (16)
C11	0.085 (3)	0.077 (3)	0.0439 (19)	0.045 (3)	0.0142 (19)	0.0162 (19)
C12	0.073 (3)	0.065 (3)	0.056 (2)	0.038 (2)	−0.007 (2)	−0.0122 (19)
C13	0.0398 (19)	0.039 (2)	0.113 (4)	0.0005 (16)	−0.009 (2)	−0.002 (2)
C14	0.046 (2)	0.070 (3)	0.071 (2)	0.0238 (19)	0.0259 (17)	0.022 (2)
C15	0.0385 (15)	0.0278 (14)	0.0277 (12)	0.0102 (12)	−0.0012 (11)	0.0034 (11)
C16	0.0454 (18)	0.0441 (19)	0.0448 (17)	0.0057 (15)	0.0059 (13)	0.0154 (14)
C17	0.074 (3)	0.061 (2)	0.0400 (17)	0.022 (2)	0.0095 (16)	0.0229 (16)
C18	0.068 (2)	0.062 (2)	0.0315 (15)	0.0255 (19)	−0.0081 (15)	0.0069 (15)
C19	0.0401 (17)	0.050 (2)	0.0470 (18)	0.0074 (15)	−0.0093 (14)	0.0025 (15)
C20	0.0413 (16)	0.0378 (17)	0.0378 (15)	0.0082 (13)	−0.0001 (12)	0.0124 (13)
O1	0.116 (2)	0.082 (2)	0.0394 (13)	0.0695 (18)	−0.0269 (14)	−0.0153 (12)
S1	0.0383 (4)	0.0438 (4)	0.0285 (3)	0.0154 (3)	−0.0040 (3)	0.0001 (3)
Br1	0.0493 (2)	0.0780 (3)	0.03575 (18)	0.01993 (18)	0.00201 (13)	−0.01404 (15)

### (II) (E)-4-(5-Bromothiophen-2-yl)-1,3-diphenylbut-3-en-2-one Geometric parameters (Å, º)

**Table:**

C1—C2	1.357 (4)	C10—C11	1.355 (5)
C1—S1	1.706 (3)	C10—H10	0.9300
C1—Br1	1.863 (3)	C11—C12	1.323 (6)
C2—C3	1.401 (4)	C11—H11	0.9300
C2—H2	0.9300	C12—C13	1.347 (6)
C3—C4	1.368 (4)	C12—H12	0.9300
C3—H3	0.9300	C13—C14	1.408 (6)
C4—C5	1.439 (4)	C13—H13	0.9300
C4—S1	1.737 (3)	C14—H14	0.9300
C5—C6	1.343 (4)	C15—C20	1.376 (4)
C5—H5	0.9300	C15—C16	1.385 (4)
C6—C7	1.489 (4)	C16—C17	1.376 (5)
C6—C15	1.494 (4)	C16—H16	0.9300
C7—O1	1.211 (4)	C17—C18	1.369 (5)
C7—C8	1.503 (4)	C17—H17	0.9300
C8—C9	1.493 (4)	C18—C19	1.363 (5)
C8—H8A	0.9700	C18—H18	0.9300
C8—H8B	0.9700	C19—C20	1.389 (4)
C9—C10	1.372 (5)	C19—H19	0.9300
C9—C14	1.377 (5)	C20—H20	0.9300
			
C2—C1—S1	113.4 (2)	C9—C10—H10	119.1
C2—C1—Br1	126.5 (2)	C12—C11—C10	120.6 (4)
S1—C1—Br1	120.05 (16)	C12—C11—H11	119.7
C1—C2—C3	111.1 (3)	C10—C11—H11	119.7
C1—C2—H2	124.5	C11—C12—C13	120.9 (4)
C3—C2—H2	124.5	C11—C12—H12	119.5
C4—C3—C2	114.5 (2)	C13—C12—H12	119.5
C4—C3—H3	122.8	C12—C13—C14	119.6 (4)
C2—C3—H3	122.8	C12—C13—H13	120.2
C3—C4—C5	125.0 (2)	C14—C13—H13	120.2
C3—C4—S1	110.0 (2)	C9—C14—C13	119.5 (3)
C5—C4—S1	124.9 (2)	C9—C14—H14	120.2
C6—C5—C4	130.1 (2)	C13—C14—H14	120.2
C6—C5—H5	114.9	C20—C15—C16	118.5 (3)
C4—C5—H5	114.9	C20—C15—C6	120.5 (2)
C5—C6—C7	116.9 (2)	C16—C15—C6	120.9 (3)
C5—C6—C15	123.1 (2)	C17—C16—C15	120.7 (3)
C7—C6—C15	120.0 (2)	C17—C16—H16	119.7
O1—C7—C6	121.4 (3)	C15—C16—H16	119.7
O1—C7—C8	121.3 (3)	C18—C17—C16	120.2 (3)
C6—C7—C8	117.3 (2)	C18—C17—H17	119.9
C9—C8—C7	115.1 (3)	C16—C17—H17	119.9
C9—C8—H8A	108.5	C19—C18—C17	120.0 (3)
C7—C8—H8A	108.5	C19—C18—H18	120.0
C9—C8—H8B	108.5	C17—C18—H18	120.0
C7—C8—H8B	108.5	C18—C19—C20	120.1 (3)
H8A—C8—H8B	107.5	C18—C19—H19	119.9
C10—C9—C14	117.5 (3)	C20—C19—H19	119.9
C10—C9—C8	121.5 (3)	C15—C20—C19	120.4 (3)
C14—C9—C8	121.0 (3)	C15—C20—H20	119.8
C11—C10—C9	121.8 (4)	C19—C20—H20	119.8
C11—C10—H10	119.1	C1—S1—C4	90.98 (13)
			
S1—C1—C2—C3	−0.8 (4)	C11—C12—C13—C14	−1.7 (6)
Br1—C1—C2—C3	178.9 (2)	C10—C9—C14—C13	−0.2 (5)
C1—C2—C3—C4	0.7 (4)	C8—C9—C14—C13	179.7 (3)
C2—C3—C4—C5	179.2 (3)	C12—C13—C14—C9	0.9 (5)
C2—C3—C4—S1	−0.3 (3)	C5—C6—C15—C20	90.7 (4)
C3—C4—C5—C6	−175.7 (3)	C7—C6—C15—C20	−90.4 (4)
S1—C4—C5—C6	3.7 (5)	C5—C6—C15—C16	−86.7 (4)
C4—C5—C6—C7	−179.0 (3)	C7—C6—C15—C16	92.2 (4)
C4—C5—C6—C15	−0.1 (5)	C20—C15—C16—C17	−0.9 (5)
C5—C6—C7—O1	−10.8 (5)	C6—C15—C16—C17	176.5 (3)
C15—C6—C7—O1	170.2 (3)	C15—C16—C17—C18	−0.8 (5)
C5—C6—C7—C8	168.2 (3)	C16—C17—C18—C19	2.0 (6)
C15—C6—C7—C8	−10.7 (5)	C17—C18—C19—C20	−1.3 (5)
O1—C7—C8—C9	6.9 (6)	C16—C15—C20—C19	1.5 (5)
C6—C7—C8—C9	−172.1 (3)	C6—C15—C20—C19	−175.9 (3)
C7—C8—C9—C10	−78.8 (5)	C18—C19—C20—C15	−0.4 (5)
C7—C8—C9—C14	101.3 (4)	C2—C1—S1—C4	0.6 (3)
C14—C9—C10—C11	0.2 (5)	Br1—C1—S1—C4	−179.13 (18)
C8—C9—C10—C11	−179.7 (3)	C3—C4—S1—C1	−0.2 (2)
C9—C10—C11—C12	−0.9 (5)	C5—C4—S1—C1	−179.7 (3)
C10—C11—C12—C13	1.7 (6)		

### (II) (E)-4-(5-Bromothiophen-2-yl)-1,3-diphenylbut-3-en-2-one Hydrogen-bond geometry (Å, º)

Cg is the centroid of the C1–C4/S1 ring.

**Table:**

*D*—H···*A*	*D*—H	H···*A*	*D*···*A*	*D*—H···*A*
C3—H3···O1^i^	0.93	2.54	3.320 (4)	141
C19—H19···*Cg*^ii^	0.93	2.90	3.768 (3)	156

Symmetry codes: (i) −*x*+1, −*y*+1, −*z*+2; (ii) *x*−1, *y*, *z*.
